# Characterization of an anterior segment organ culture model for open globe injuries

**DOI:** 10.1038/s41598-021-87910-8

**Published:** 2021-04-20

**Authors:** Eric J. Snider, Emily N. Boice, Brandon Gross, Jacinque J. Butler, David O. Zamora

**Affiliations:** Department of Sensory Trauma, United States Army Institute of Surgical Research, Fort Sam Houston, TX 78234 USA

**Keywords:** Experimental models of disease, Corneal diseases, Biomedical materials

## Abstract

Open-globe injuries have poor visual outcomes and have increased in frequency. The current standard of care is inadequate, and a therapeutic is needed to stabilize the injury until an ophthalmic specialist is reached. Unfortunately, current models or test platforms for open-globe injuries are insufficient. Here, we develop and characterize an open-globe injury model using an anterior segment organ-culture platform that allows therapeutic assessment for up to 72 h post-injury. Anterior segments maintained in organ culture were kept at physiological intraocular pressure throughout, and puncture injuries were created using a novel pneumatic-powered system. This system can create high-speed, military-relevant injuries up to 4.5 mm in diameter through the cornea. From intraocular pressure readings, we confirmed a loss of pressure across the 72 h after open-globe injury. Proof-of-concept studies with a Dermabond tissue adhesive were performed to show how this model system could track therapeutic performance for 72 h. Overall, the organ-culture platform was found to be a suitable next step towards modeling open-globe injuries and assessing wound closure over the critical 72 h post-injury. With improved models such as this, novel biomaterial therapeutics development can be accelerated, improving care, and, thus, improving the prognosis for the patients.

## Introduction

Open-globe (OG) injuries have increased in frequency in recent combat operations and are expected to continue to rise due to further reliance on explosive weaponry^1^. Currently, OG injuries have poor visual outcomes for the injured warfighter, often resulting in irreversible blindness^2^. The current standard of care is inadequate. If an OG injury is suspected on the battlefield, a rigid eye shield is applied to protect the eye, and the injured warfighter is evacuated to an ophthalmic specialist. Then, OG injuries are closed with sutures to create a watertight seal. However, this process may occur up to 24 h post-injury currently and is expected to increase up to 72 h in future combat operations where air evacuation may not be guaranteed^3–5^. In addition, delay is prevalent in remote parts of the world where ophthalmic care is not accessible for many days after injury^6,7^. This delay between injury and sealing the OG injury can negatively impact the long-term prognosis. An untreated OG injury is highly susceptible to infection, and reduced intraocular pressure (IOP) impacts ocular function in addition to tissue viability^8–10^. If therapeutics can be applied more rapidly after OG injury to seal the injury site, the long-term prognosis for the patient may be improved.

Towards this, we recently developed a benchtop OG injury model as a first step towards replicating OG injuries in fresh porcine tissue^11,12^. OG injuries were created using a high-speed computer-controlled solenoid device, creating large, irregularly shaped corneal puncture wounds. Further, we have utilized this model to begin assessing the stabilization of these wounds using off-the-shelf therapeutics, such as Dermabond tissue adhesive^13^. While this was an ideal platform for first steps, it requires the use of whole porcine eyes. As many of the posterior tissues of the eye are vascularized and rapidly degrade post-mortem, this model limits assessment of therapeutic testing for only a few hours post-injury. However, proper combat casualty care stipulates the need for stabilizing the injury site for up to 72 h^3–5^. Successfully sealing an OG injury immediately post-injury, while an important first step, does not necessarily correlate to strong performance over the 72 h window. As a result, an OG injury platform is needed for assessing wound closure across this critical 3 day window.

Towards this, here, we have utilized an anterior segment organ culture (ASOC) platform for maintaining ocular tissue during this critical 72 h window. ASOC is a widely used technique for maintaining avascular anterior segment tissues for multiple weeks post-enucleation^14–18^. The corneoscleral shell is preserved for ASOC, along with the trabecular meshwork outflow region as it is responsible for regulating IOP^19–21^. Tissue is maintained under physiological IOP throughout while clamped in the ASOC platform, and this system has been widely used for studying conditions such as glaucoma and corneal trauma as well as cell and pharmaceutical based therapeutics^17,18,22–24^. Further, ASOC can be advantageous compared to animal testing in some situations as it can be configured for high-throughput assessment with rabbit, porcine, monkey, or even human tissue^14–18,22–24^. Here, we show how a pneumatic-powered puncture device can be utilized in an ASOC platform to create an OG injury model. In addition, we demonstrate through a proof-of-concept study how this model can be utilized for assessing efficacy of potential wound sealing products across the critical 72 h after OG injury.

## Results

### Anterior segment organ culture setup

**Figure 1:**
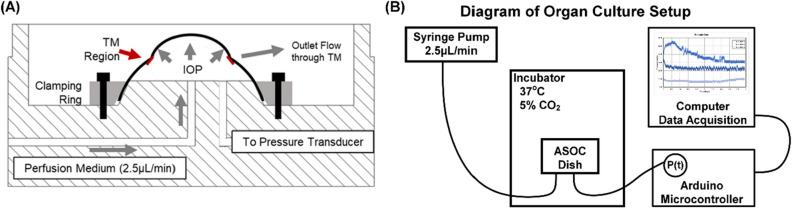
Overview of anterior segment organ culture setup. (**A**) Diagram of ASOC dish and clamping ring. (**B**) Diagram of the organ culture setup. Syringe pump delivers organ culture media at physiological flow rates to the ASOC setup that is kept in an incubator at 37 °C, 5% CO_2_. Additional fluidic line attached to pressure transducer records data with a microcontroller and computer in real-time. Figure 1A is reproduced from Snider et al., 2019.

**Figure 2:**
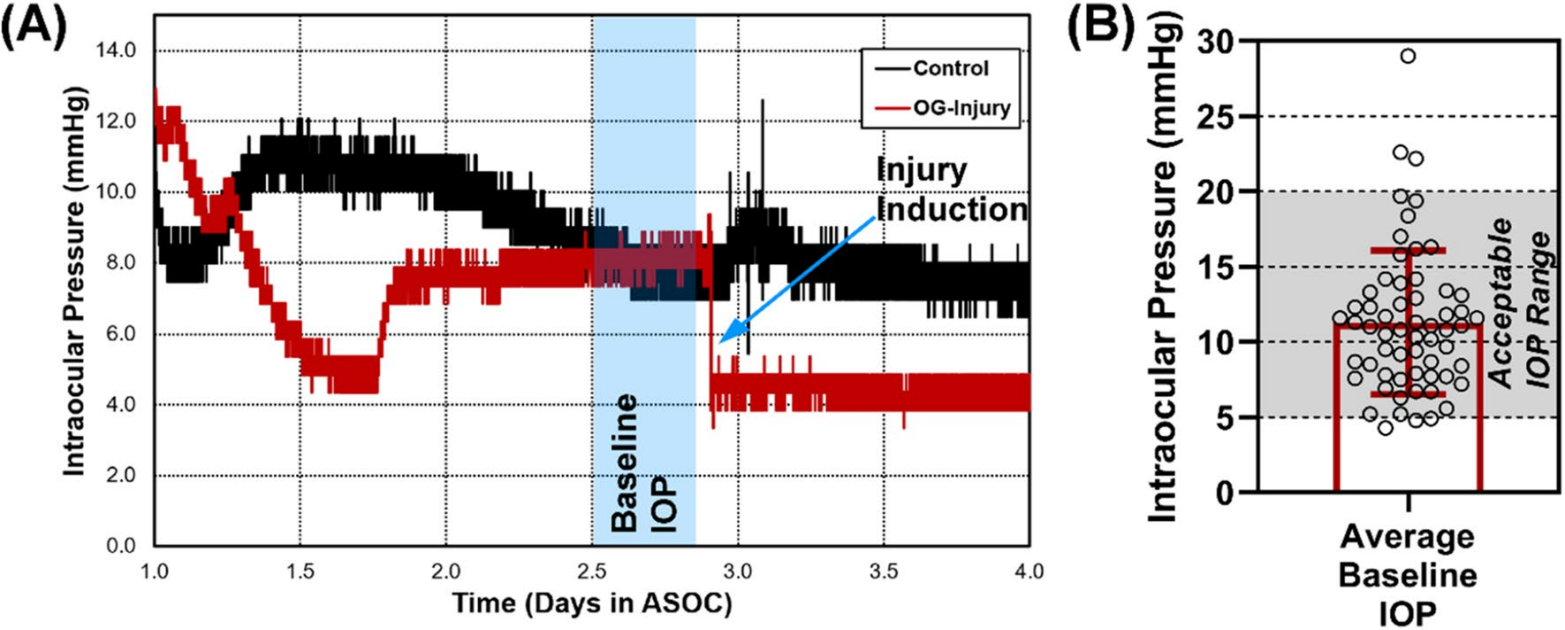
Intraocular pressure results for anterior segment organ culture. (**A**) Representative data showing IOP traces across the ASOC setup. Region where IOPs were averaged to determine baseline IOP, defined as the region immediately prior to OG injury, is shown. (**B**) Stabilized IOP for all ASOC experiments, with 6 experiments falling outside the acceptable IOP range, 5 to 20 mmHg^17^.

Prior to introduction of OG injuries in ocular tissue, we first evaluated the effect of ASOC on corneal properties. This established a baseline of comparison for injury induction experiments and an understanding for how tissue was impacted by the culture setup. The ASOC platform made use of custom-fabricated dishes and clamping rings to secure the corneoscleral shell (Fig. 1A). Media was perfused by syringe pump into the anterior segment (AS) at physiological flow rates and IOP was recorded by pressure transducers attached to each ASOC dish (Fig. 1B). IOP data was collected in real time throughout experiments, and IOP stabilized approximately 2–3 days after setup, similar to previous ASOC studies (Fig. 2A)^17,18^. Average stabilized IOP prior to injury induction was used as a baseline and was approximately 11 mmHg in all eyes tested (Fig. 2B). Eyes were removed from consideration if their stabilized IOP prior to injury induction was below 5 mmHg or above 20 mmHg^17,18^. This criterion resulted in a 10% ASOC exclusion rate (6 eyes out of 60 total tested).

### Effect of ocular tissue maintained in anterior segment organ culture

**Figure 3:**
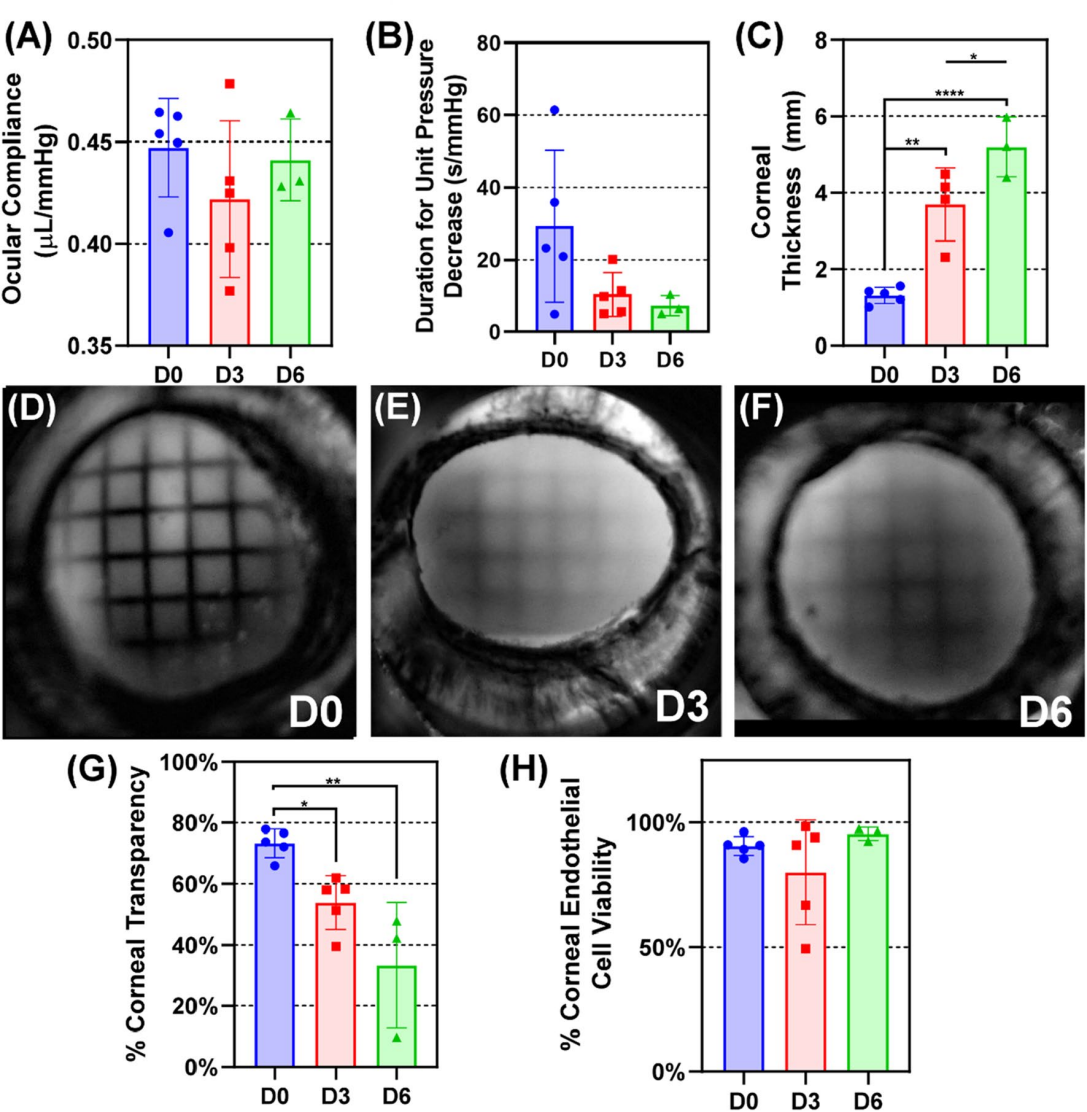
Assessment of corneal properties for AS tissue maintained in OC. (**A**) Ocular compliance for control D0, D3, D6 data. (**B**) Calculated durations per unit IOP from 18 to 15 mmHg for D0, D3, and D6 uninjured, control tissue. (**C**) Quantification of corneal thickness for uninjured tissue. (**D-F**) Representative processed images for (**D**) D0, (**E**) D3, and (**F**) D6 control eyes for assessing transparency. (**G**) Quantification of corneal transparency. (**H**) Quantification of tissue viability. Results are shown as mean and error bars denote standard deviation throughout. Asterisks denote (*) *p* < 0.05, (**) *p* < 0.01, (***) *p* < 0.001, and (****) *p* < 0.0001 as determined by one-way ANOVA, post-hoc Tukey’s test.

Control eyes were removed from culture immediately after ASOC setup (D0), after 3 days in culture (D3, when OG injuries will be induced in subsequent groups), or after 6 days (D6, equivalent timepoint for 72 h post-injury) for use as uninjured baselines to compare to injured eyes. We first looked at ocular compliance, a lumped mechanical property describing how intraocular pressure changes due to inflation (change in volume/change in pressure). D0 results trended on average above D3 and D6 groups, but the difference was not statistically significant (Fig. 3A). Next outflow rate was assessed by finding the linear slope of IOP (mmHg) vs. Time (seconds) while eyes were set at 18 mmHg and tracked until IOP fell to 15 mmHg. Trends were evident for outflow rate measurements, with faster rates occurring at D3 and D6 timepoints (Fig. 3B). However, these differences were not significant. One major change occurring in organ culture was corneal swelling, with the tissue significantly thickening at D3 and D6 compared to D0, as well as significantly thickening between D3 and D6 (Fig. 3C). Corneal transparency was quantified by measuring the grid area visible through the cornea (Fig. 3D–F). There was a significant reduction in transparency at D3 and D6 when compared to D0 results, but differences between D3 and D6 were not significant (Fig. 3G). Lastly, since the corneal endothelium is one of the most sensitive cell layers of the cornea and responsible for maintaining corneal clarity, viability of the corneal endothelium was assessed from live-dead staining and captured by confocal microscopy. Overall, corneal endothelium viability was similar across OC experiments and remained close to 100% (Fig. 3H).

### Characterization of anterior segment after open-globe injury

**Figure 4:**
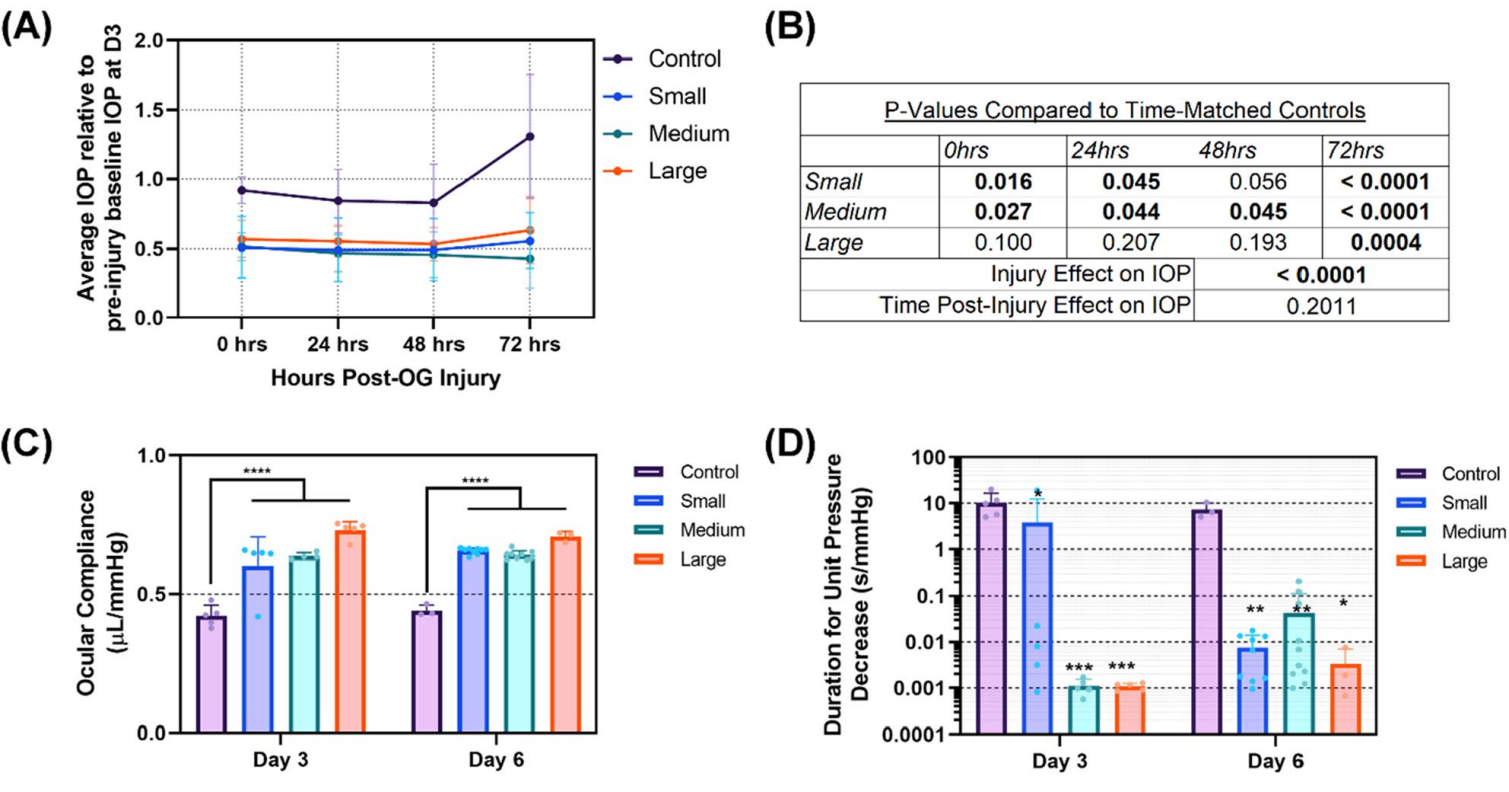
Mechanical property changes after OG injury in ASOC injury platform. (**A**) Average IOP relative to baseline levels immediately after injury and 24, 48, and 72 h post injury for control, small, medium, and large injury sizes. (**B**) Summary of statistical difference for IOP data for each injury state vs. its time matched control. *p* values for the injury and time effect on IOP are also shown, as determined by 2-way ANOVA. (**C**) Ocular compliance and (**D**) calculated durations per unit IOP from 18 to 15 mmHg for control, small, medium, large OG injury sizes immediately post injury (D3 organ culture) and 3 days post injury (D6 organ culture). Durations are logarithmically plotted to better show large and small values on a single plot. Results are shown as mean and error bars denote standard deviation throughout. Asterisks denote (*) *p* < 0.05, (**) *p* < 0.01, (***) *p* < 0.001, and (****) *p* < 0.0001 as determined by two-way ANOVA, post-hoc Dunnett’s test.

Next, we compared the effect of three injury sizes on the ASOC model: small (1.2 mm Diameter), medium (2.4 mm D), and large (4.5 mm D). Injuries were induced using a custom pneumatic powered device at 50 psi (~ 2600 mmHg, see “Pneumatic Open Globe Injury Device” for more details). Injuries were created on Day 3 of ASOC and removed for characterization immediately (noted as D3, relative to ASOC setup, not injury) or 72 h post OG injury (noted as D6). We first tracked changes in IOP following OG injury for the 72 h after injury to compare to baseline IOP before injury (Fig. 4A). The effect of the injury on IOP resulted in a significant reduction in IOP (*p* < 0.0001) as determined by two-way ANOVA (Fig. 4B). Further, most injury size IOPs were significantly different from control data at all time points (Fig. 4B). There was not a significant effect of time on IOP post-injury, however. This confirms the induced OG injuries consistently resulted in loss of the water-tight seal of the cornea, resulting in loss of physiological IOP. Furthermore, the significant IOP reductions were still evident at 72 h, suggesting all injury sizes tested were not capable of full wound closure in this critical timeframe.

**Figure 5:**
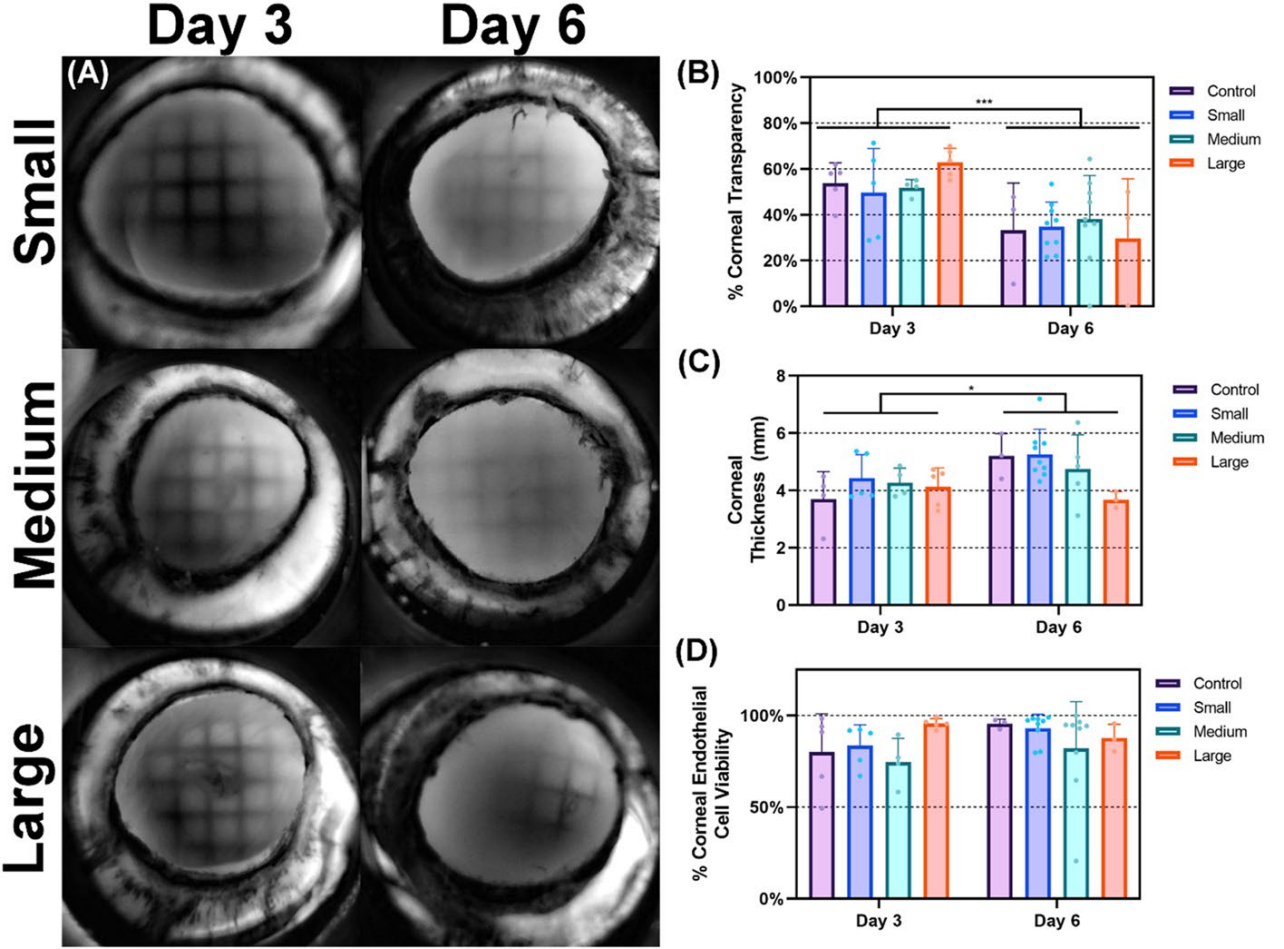
Assessment of OG injury corneal transparency, thickness, and viability. (**A**) Representative transparency images for small (first row), medium (second row), and large (Third Row) injuries D3 (Left Column) and D6 (Right Column). (**B**) Cell transparency, (**C**) corneal thickness, and (**D**) corneal endothelium viability for control, small, medium, large OG injury sizes immediately post injury (D3 organ culture) and 72 h post injury (D6 organ culture). Results are shown as mean and error bars denote standard deviation throughout. Asterisks denote (*) *p* < 0.05, (**) *p* < 0.01, (***) *p* < 0.001, and (****) *p* < 0.0001 as determined by two-way ANOVA, post-hoc Dunnett’s test.

Ocular compliance was significantly altered following OG injury, but the size of the injury had a minimal effect. In addition, changes to ocular compliance between D3 and D6 timepoints were insignificant (Fig. 4C). Pressure drops during the outflow rate tests were nearly all instantaneous and all durations for each injury size were significantly decreased compared to time-matched controls (Fig. 4D). This is consistent with IOP results and indicative of successful open globe injury induction. Both, corneal thickness and transparency were significantly impacted by time, similar to control studies but the injury itself did not cause a change (Fig. 5 A–C). Lastly, corneal endothelial viability was not altered by OG injury, in comparison to time-point matched control tissue (Fig. 5 D).

**Figure 6:**
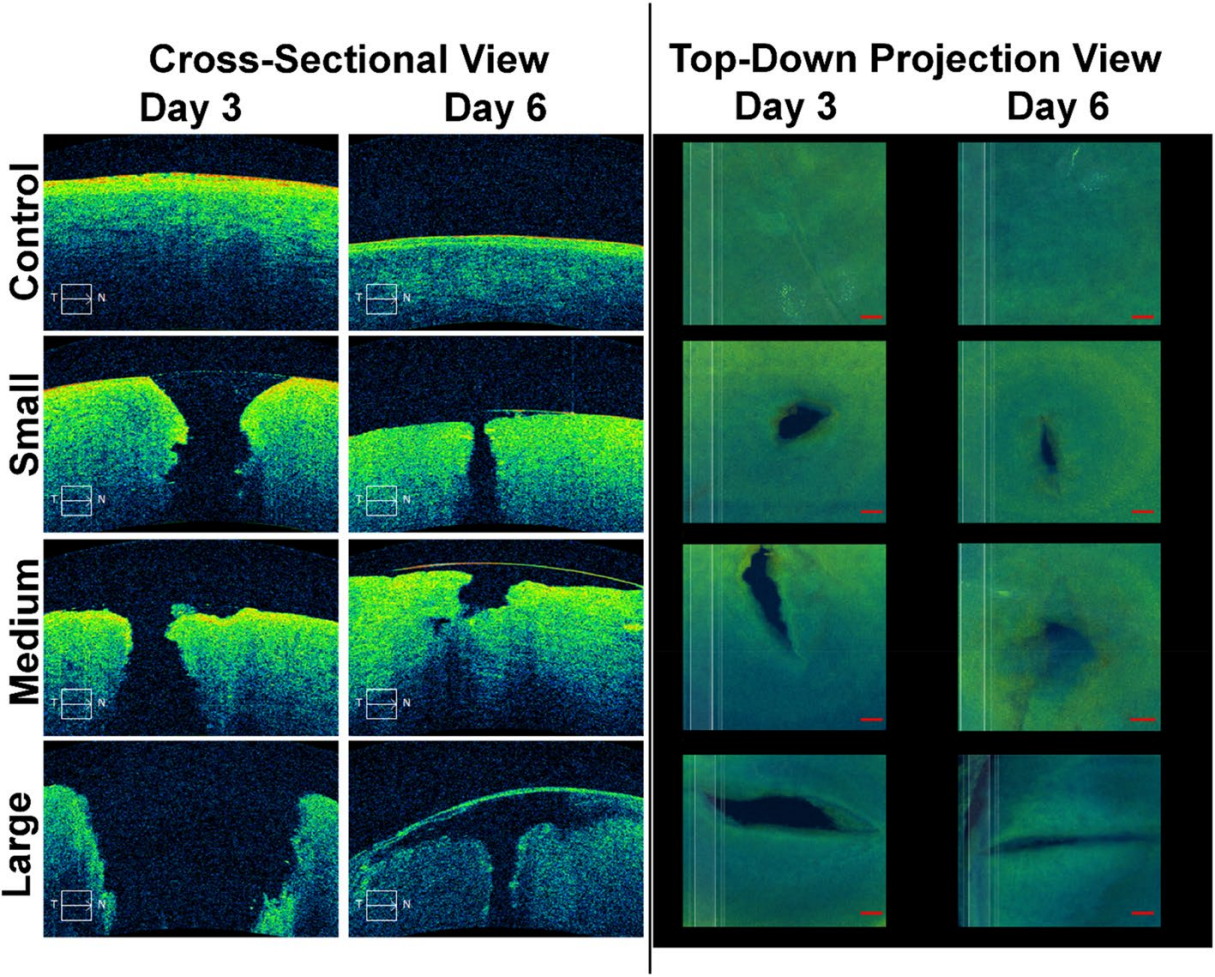
OCT analysis of OG injuries. Representative (left columns) cross-sectional and (right columns) top-down images of the cornea for control, uninjured (row 1), small (1.2 mm D, row 2), Medium (2.4 mm D, row 3), and Large (4.5 mm D, row 4) OG injuries. Images were taken immediately after OG injury (D3, column 1) and 72 h post injury (D6, column 2). Scale denoted by 0.3 × 0.3 mm square in corner of images (Left column) or 0.3 mm scale bar (right column).

**Figure 7:**
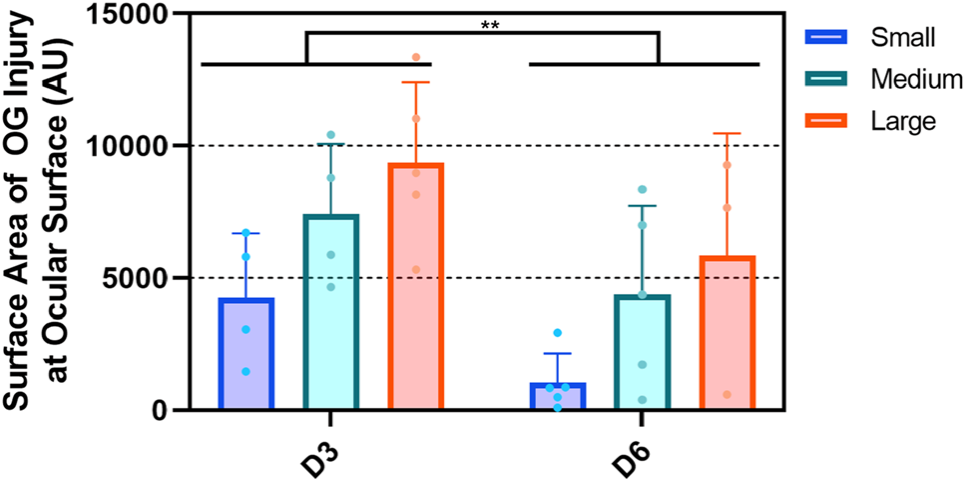
Injury surface area quantification from OCT scans. Results for small, medium, large OG injury size are shown immediately post injury (D3) and 72 h post injury (D6). Data are shown as mean value with error bars denoting standard deviation. Asterisks denote (*) *p* < 0.05, (**) *p* < 0.01, (***) *p* < 0.001, and (****) *p* < 0.0001 as determined by two-way ANOVA, post-hoc Šídák method.

Eyes were further evaluated by optical coherence tomography (OCT) to better visualize the OG injury in the cornea. Cross sectional views through the corneal tissue revealed large injury sizes that scaled with the OG injury size. However, the injury was inconsistent in shape and diameter through the cornea, making quantification of cross sectional images challenging (Fig. 6, Right Columns). A top-down projection of the cornea represented an easier to quantify injury surface area (Fig. 6, Left Columns). Due to irregular shape, injury surface area was variable between replicates, but results trended directly with the diameter of the puncture object (Fig. 7). Interestingly, a reduction in OG injury size was evident in all later time points compared to D3 results. While each injury size area was not significantly reduced from D3 to D6 due to high variability, time post-injury was identified as a significant point of variation (*p* < 0.01) as was injury size (*p* < 0.05).

### Effect of tissue adhesive on sealing open globe injury

**Figure 8:**
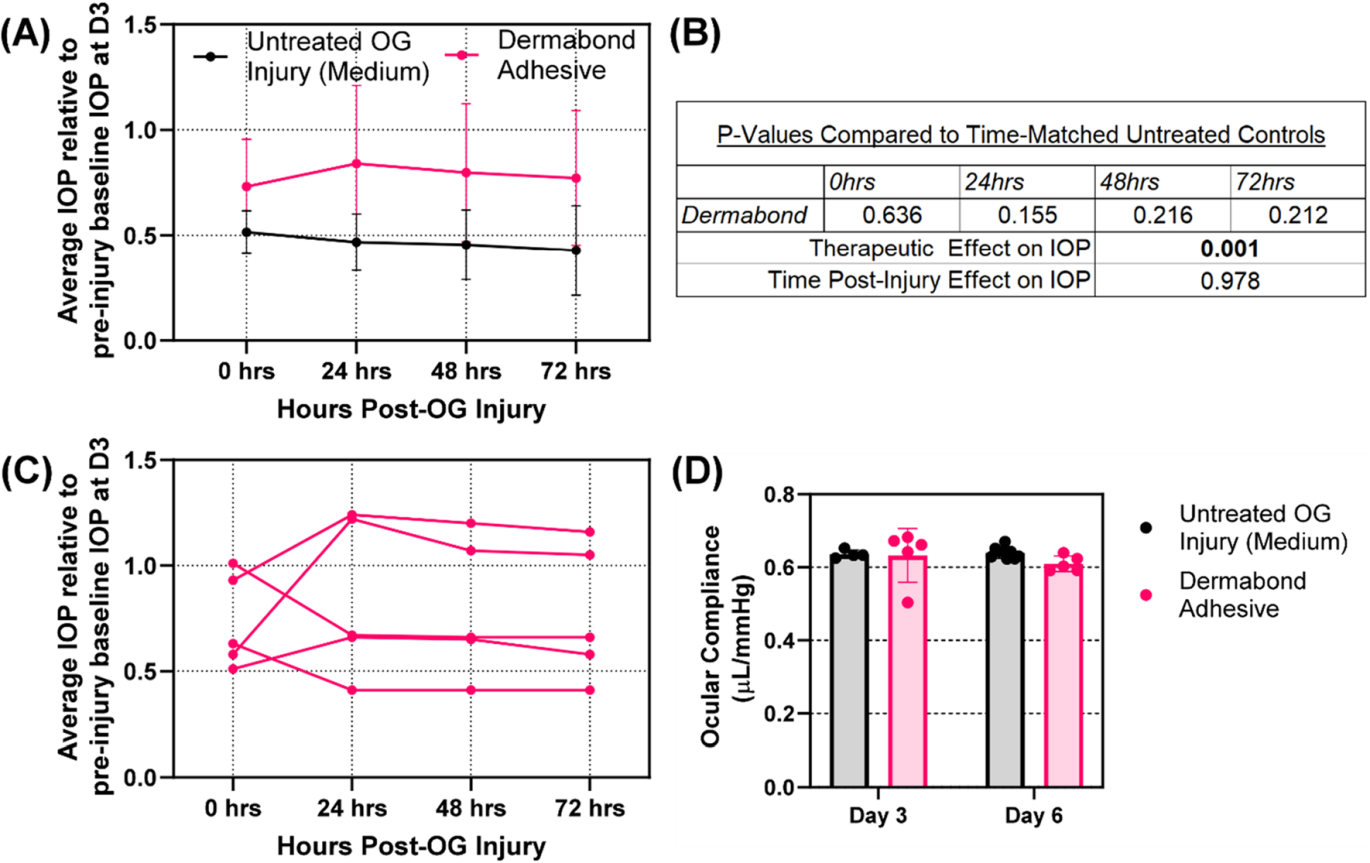
Effect of Dermabond tissue sealant on maintaining IOP and Ocular compliance. (**A**) Average IOP relative to baseline levels immediately after injury and 24, 48, and 72 h post injury for untreated and Dermabond treated tissue. (**B**) Summary of *p* values for Dermabond vs. untreated time-matched controls, as well as the effect of therapeutic and time on IOP, as determined by two-way ANOVA, post-hoc Šídák method. Values are bolded when statistically significant (*p* < 0.05) (**C**) Each of 5 Dermabond treated replicate responses across the 72 h following injury and treatment to highlight the variability between the replicates. (**D**) Ocular compliance for untreated and Dermabond treated tissue immediately post injury (D3 organ culture) and 72 h post injury (D6 organ culture). Results are shown as mean and error bars denote standard deviation throughout.

A pilot study was then performed with Dermabond tissue adhesive applied as a candidate therapeutic to seal OG injuries across the critical 72 h window. First, we looked at the impact of tissue adhesive on restoring IOP after it was applied to an open globe injury (Fig. 8A). For simplicity in this pilot study, a single medium injury size was assessed. Differences at each time point between untreated and treated conditions were not significant, but there was a significant effect due to therapeutic on IOP (Fig. 8B). There was a clear trend in IOP recovery to near baseline levels after adhesive application, however variation was high after wound closure, indicating high variability in the application of the adhesive to the eye. This is made more evident by looking at the five replicate Dermabond treated experiments separately, which makes it clear that two cases had strong IOP recovery while three did not recover (Fig. 8C).

**Figure 9:**
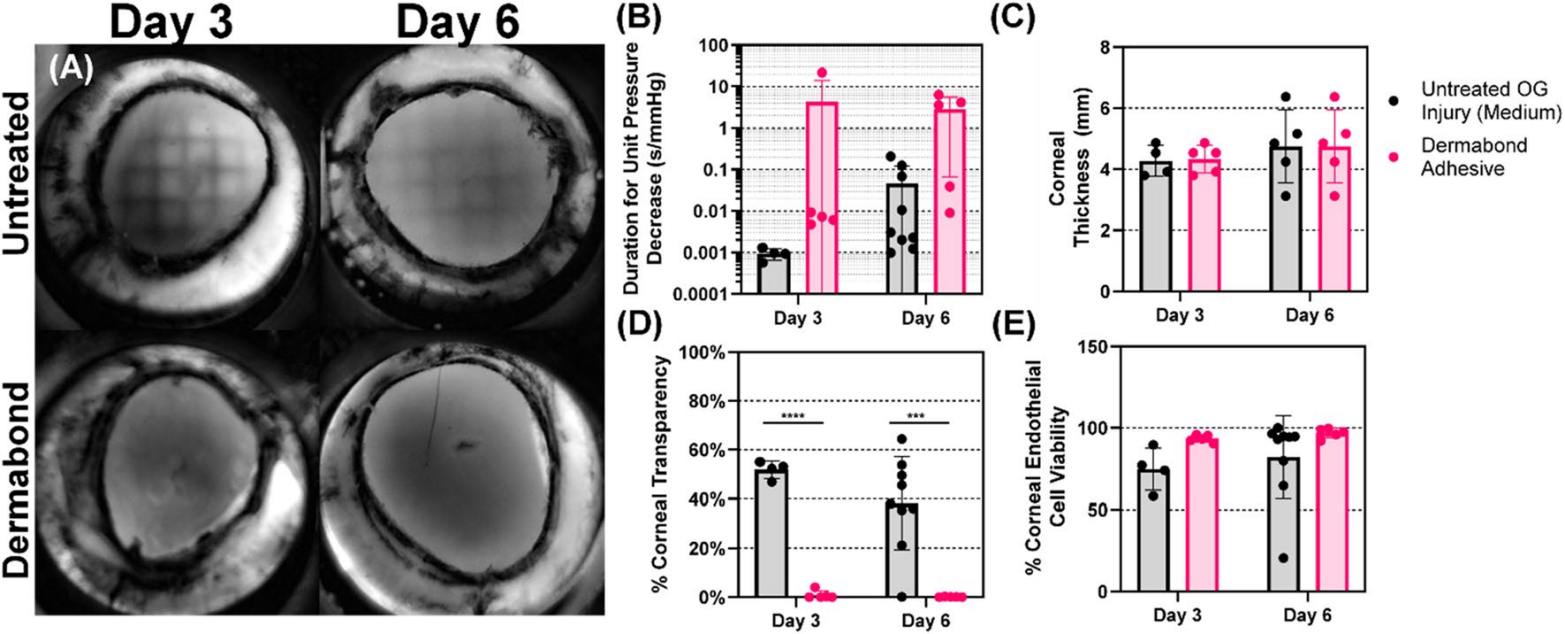
Assessment of Dermabond adhesive on open globe injuries. (**A**) Representative transparency images of medium OG injured (top row) and Dermabond treated (bottom row) eyes at D3 (left column) and D6 (right column). (**B**) Outflow duration (**C**) corneal thickness, (**D**) corneal transparency and (**E**) cell viability for untreated and Dermabond treated tissue immediately post injury (D3 organ culture) and 72 h post injury (D6 organ culture). Results are shown as mean and error bars denote standard deviation throughout. Asterisks denote (*) *p* < 0.05, (**) *p* < 0.01, (***) *p* < 0.001, and (****) *p* < 0.0001 as determined by two-way ANOVA, post-hoc Šídák method.

Ocular compliance (Fig. 8D) and corneal thickness (Fig. 9C) were minimally impacted by the adhesive treatment, but outflow rate calculations (Fig. 9B) had a noticeable but statistically insignificant increase in outflow durations for both time points, indicating proper wound sealing in some replicates. Interestingly, cell viability was significantly increased after Dermabond treatment when compared to untreated eyes, however, the magnitude of the difference was minimal (Fig. 9E). Conversely, there was a major impact on corneal transparency, with the Dermabond treatment resulting in fully opacified corneal tissue at both time points (Fig. 9A,D).

## Discussion

Currently, OG injuries have poor vision prognosis for the injured warfighter as well as the civilian population. Unfortunately, OG injuries are increasing in prevalence due to further reliance on explosive weaponry. The current standard of care is inadequate and often results in an OG injury remaining untreated for up to 24 h. This is anticipated increasing to as much as 72 h for future operations. This delay between treatment and injury is hypothesized to be a major contributing factor to the poor visual outcome following OG injury as an untreated injury could result in infection and loss of tissue viability. In order to develop therapeutics, pathophysiologically relevant injury models are required. Here, we develop and characterize an OG anterior segment organ culture (ASOC) injury model that is capable of assessing therapeutic performance across this critical 72 h window post-OG injury.

ASOC is a widely used technique for maintaining anterior tissues of the eye, such as the cornea, for many days and was thus an ideal platform for this injury model^15–17^. The 72 h window after OG injury critical to military medicine was believed to be trackable with this approach. Porcine eyes are similar in size to human, so military-relevant injuries were possible. This platform provided a simplified testing platform prior to in vivo animal testing. Injury progression was characterized across a wide range of metrics (such as IOP stability, corneal thickness and transparency, etc.). IOP is essential for maintaining intraocular tissues and a loss of IOP can indicate a loss of the watertight seal of the eye. Watertight seal integrity is essential for preventing intraocular infection on the battlefield and was further measured by outflow rate testing. Changes in tissue mechanical properties were measured by ocular compliance. Corneal edema was assessed by corneal transparency and thickness assessment. Corneal edema, in the short term, may result in an inability to properly assess intraocular damage or detect foreign bodies, and, in the long term, may require a corneal transplant. Lastly, corneal endothelium viability was measured to determine the effect of ASOC and OG injury on 72 h corneal health.

We were successfully able to create OG injuries with the ASOC platform. IOP stabilized after 3 days in culture and the pneumatic linear actuation-based puncture device was successful at creating injuries up to 4.5 mm in size. Interestingly, corneal endothelial viability and overall corneal transparency were not significantly altered after OG injury compared to control tissue. There was an increase in opacity, but this was at the same rate as control tissue. The OG injury should lead to disruption of the corneal endothelium, exposing more stromal tissue to potential edema. However, it is possible the large changes in transparency and corneal thickness occurring in control tissue may dwarf or hide OG injury-related changes. Conversely, IOP dropped significantly following OG injury and remained as such across the 72 h post injury window for all injury sizes. Of note, IOP was similarly reduced for all injury sizes.

From OCT imaging, a reduction in the surface area of the OG injury was observed after injury induction. From cross sectional views of the cornea, wound closure was more evident at the epithelium surface. Corneal wound healing is a complex process involving a cascade of molecular pathways when even a single layer is damaged but is further complicated by a full thickness injury such as this^25^. In single zone corneal injuries, initial epithelium wound closure is on the order of magnitude of 72 hours^26^ while stromal defects can take up to a week^27^. Unfortunately, due to the nature of the ASOC setup, blood derived factors known to be involved in the fibrotic wound healing process are missing which could slow or fully inhibit proper wound closure^28^. Another confounding factor in this is the swelling of the cornea which may give the impression of reduced injury size. While injury size was reduced, none of these injuries reached full closure as IOP remained low after 72 h. It is possible that by tracking injury progress beyond 72 h or with smaller injuries, full wound closure may be seen. Identifying an injury size capable of self-healing across the 72 h window would be valuable insight for combat medics and ophthalmologists, and future work identifying this critical size is needed. In addition, studies looking at molecular pathways and evidence of wound healing are required.

We next showed proof-of-concept for how this platform could be utilized for evaluating potential therapeutics. With this model, we can create injuries in anterior segment tissue, apply therapeutic, and track changes across the key 72 h window where a temporary therapeutic is desperately needed for patient care. Our example utilized Dermabond, as we have shown it could seal a wide range of OG injury shapes and sizes with our benchtop OG injury platform^13^. Biomaterial-, cell-, or pharmaceutical-based therapeutics could all be easily assessed similarly, and slight modifications of the test platform could allow for a wider range of testing. First, treatment could be delayed for a more realistic wound closure scenario as a medic is unlikely to apply therapeutic immediately following injury but, more realistically, hours after the injury. Second, the ASOC platform is readily adaptable for use with human donor tissue which could allow for critical testing to support clinical trials. Lastly, longer time points beyond 72 h can be evaluated as OC has been used for maintaining anterior tissue for weeks^24^. This could be critical for assessing wound-healing and regenerative therapeutics for fully combating OG injuries.

While Dermabond was utilized in a proof-of-concept manner for showing the potential of the ASOC platform, this work clearly showed the potential for Dermabond as a tissue sealant for OG injuries. Dermabond was applied to 2.4 mm injuries immediately following injury, and there was a significant recovery in IOP compared to untreated eyes. Average IOP was unable to recover to uninjured baseline levels, but this was due to a key limitation in Dermabond: inconsistent application to the injury site. Dermabond is a liquid sealant when applied, and unfortunately the concave structure of the eye and high degree of curvature at the corneal apex resulted in the majority of the sealant not remaining on the injury site. Excessive amounts were applied to combat this, but this resulted in an inconsistent amount of sealant remaining on the injury site, resulting in variability results across the ten replicates (n = 5 D3, D6). This was further exemplified in OCT results in which the Dermabond applied to the corneal surface was widely inconsistent. Apart from loss of corneal transparency, Dermabond had promising results, but a means of improving application to the cornea is needed so that more consistent wound sealing is possible. As for the loss of transparency, the question remains as to whether this alteration was due to Dermabond opacity or permanent alterations to the cornea. Studies are needed looking at removal of Dermabond at 72 h post injury and determining if the loss of transparency is reversible.

Overall, the anterior segment organ culture platform was successfully adapted for use with OG injury studies, however, there are limitations with this approach as it currently stands. First, while the ASOC platform maintains tissue under physiological conditions, vascularized tissues and the entire posterior segment are removed. It is likely that the iris and lens, which are both removed in this model, will be damaged during the puncture process and may play a role in confounding OG injury wound healing. Further, the acceptable IOP range of 5 to 20 mmHg is wider than physiological IOP values as the ASOC system is variable in setup and designed for high-throughput testing. Next steps for this project will focus on adapting this OG injury model for use with in vivo studies. Second, while ASOC tissue remained viable across the 6 day time course, there was significant corneal swelling that resulted in loss of transparency without injury induction, which is a non-physiological confounding factor for these studies. Other ASOC preparations have observed preservation of transparency for at least 1 week, so modifications to the injury model are required to resolve this issue^29^. In addition previous work has shown that agents can be added to change the osmolarity of the OC perfusion media as a means of combatting this problem^30,31^. However, these agents may impact the trabecular meshwork and result in less-physiological IOP.

Third, we have only tested the platform out to 6 days post-initial setup. While timepoints out to at least 14 days were possible in other ASOC applications, it is unclear if additional physiological challenges will occur if OG injuries are tracked out to later timepoints. Lastly, while a range of smaller puncture objects and a range of injury shapes is possible, the maximum size for the ASOC platform is likely close to the 4.6 mm diameter size tested in this study. This is due to the force required to puncture the eye, as the 4.6 mm object almost strikes the floor of the ASOC dish behind the cornea in order to create an injury. A large object size would likely not be able to create a successful puncture without redesigning the ASOC dish or significantly increasing the injury induction pressure.

In conclusions, we have shown that an OG injury model can be developed using an anterior segment organ culture platform. With this platform we can mimic physiological IOP in porcine eyes, create an OG injury, and track changes at least 72 h post-injury. This 72 h window is of critical importance to military medicine as future battlefield scenarios may make it impossible to evacuate an OG-injured warfighter for up to 3 days. Thus, it is critical to develop a therapeutic that may stabilize the eye during this window. A wide range of OG injury sizes were tested and the pneumatic puncture device was able to consistently create injuries in corneal tissue maintained in organ culture. Furthermore, Dermabond therapeutic was tested for its ability to seal an OG injury as proof of concept of the potential for this injury platform. Results showed that Dermabond was successful at sealing OG injuries but in its current formulation application to the injury site was highly variable. Future work will begin testing novel biomaterial therapeutics for more consistently sealing OG injuries for up to 72 h post-injury. Overall, this is a critical next step towards developing a temporary wound repair product for OG injuries and improving the long-term vision prognosis for the injured patients.

## Methods and materials

### Tissue sourcing

Porcine eyes (Animal Technologies, Tyler, TX, USA) were obtained within 24 h of enucleation. Eyes remained on ice and in phosphate buffered saline (PBS, Thermo Fisher Scientific, Waltham, MA, USA) throughout shipment and until use. Extraorbital connective tissue was dissected leaving only the globe and optic nerve intact. Tissue was immersed in PBS containing 1 × penicillin, streptomycin, and amphotericin (PBS-PSA, Thermo Fisher Scientific, Waltham, MA, USA) for a minimum of 20 min to minimize the contamination risk throughout organ culture experiments. After 20 min, eyes were transferred into sterile, fresh PBS-PSA within a laminar flow cabinet.

### Preparation of ocular tissue

Anterior segment (AS) tissue was prepared as previously described^15–18^. Briefly, under aseptic conditions, eyes were hemisected using a scalpel and curved Vannas scissors to isolate the anterior segment. Vitreous humor was removed with curved scissors. Under a dissecting microscope, the lens, iris, ciliary body and other vascularized tissues were carefully removed, leaving the corneoscleral shell and trabecular meshwork tissue (TM). The TM naturally filters aqueous humor from the anterior chamber, and the resistance across this tissue and underlying tissues are responsible for producing an intraocular pressure, essential for maintaining anterior tissue under physiological conditions^19^. A cotton applicator was used to wipe the inner scleral surface and carefully touch the corneal endothelium to remove any remaining pigmented residue which could clog the TM. AS tissue was transferred to Dulbecco’s modified Eagle’s medium (Thermo Fisher Scientific, Waltham, MA, USA) containing 4% fetal bovine serum (Thermo Fisher Scientific, Waltham, MA, USA), 2 mM L-glutamine (Thermo Fisher Scientific, Waltham, MA, USA), and 1 × PSA (ASOC media) until assembly in culture dishes^29^.

### Anterior segment organ culture model

ASOC is a widely used technique for maintaining AS tissue for weeks ex vivo^14–17^. Computer-aided designs (Autodesk Inventor, San Rafeal, CA, USA) for ASOC dishes and clamping rings were generated and parts were fabricated from polycarbonate by CNC milling (MDX-50, Roland DGA, Irvine, CA, USA and Nomad 883 Pro, Carbide 3D, Torrance, CA, USA). Briefly, the ASOC dish clamps the peripheral scleral tissue with a clamping ring and stainless-steel screws (Fig. 1A). Small diameter polyethylene tubing (Becton Dickinson, Franklin Lakes, NJ, USA) was connected to stainless-steel connectors in the OC dish to deliver ASOC media into the AS by syringe pump (Harvard Apparatus, Holliston, MA, USA) at physiological aqueous humor inflow rates (2.5µL/min)^17,18^. ASOC dishes were placed in incubator held at 37 °C and 5% CO_2_. A second connection port was attached to a pressure transducer (Honeywell International, Charlotte, NC, USA) to record intraocular pressure (IOP) continuously (1 reading/10 s), via microcontroller (MEGA 2560, Arduino, Somerville, MA, USA) and computer data acquisition (Fig. 1B). The organ culture system built for this work allowed for continuous ASOC media fluid flow and IOP data acquisition for 10 ASOC setups simultaneously. Due to scleral tissue compression, all ASOC clamping rings were re-tightened 24 h after initial setup, and any residual air bubbles trapped in the AS were flushed at this time.

### Pneumatic open globe injury device

**Figure 10:**
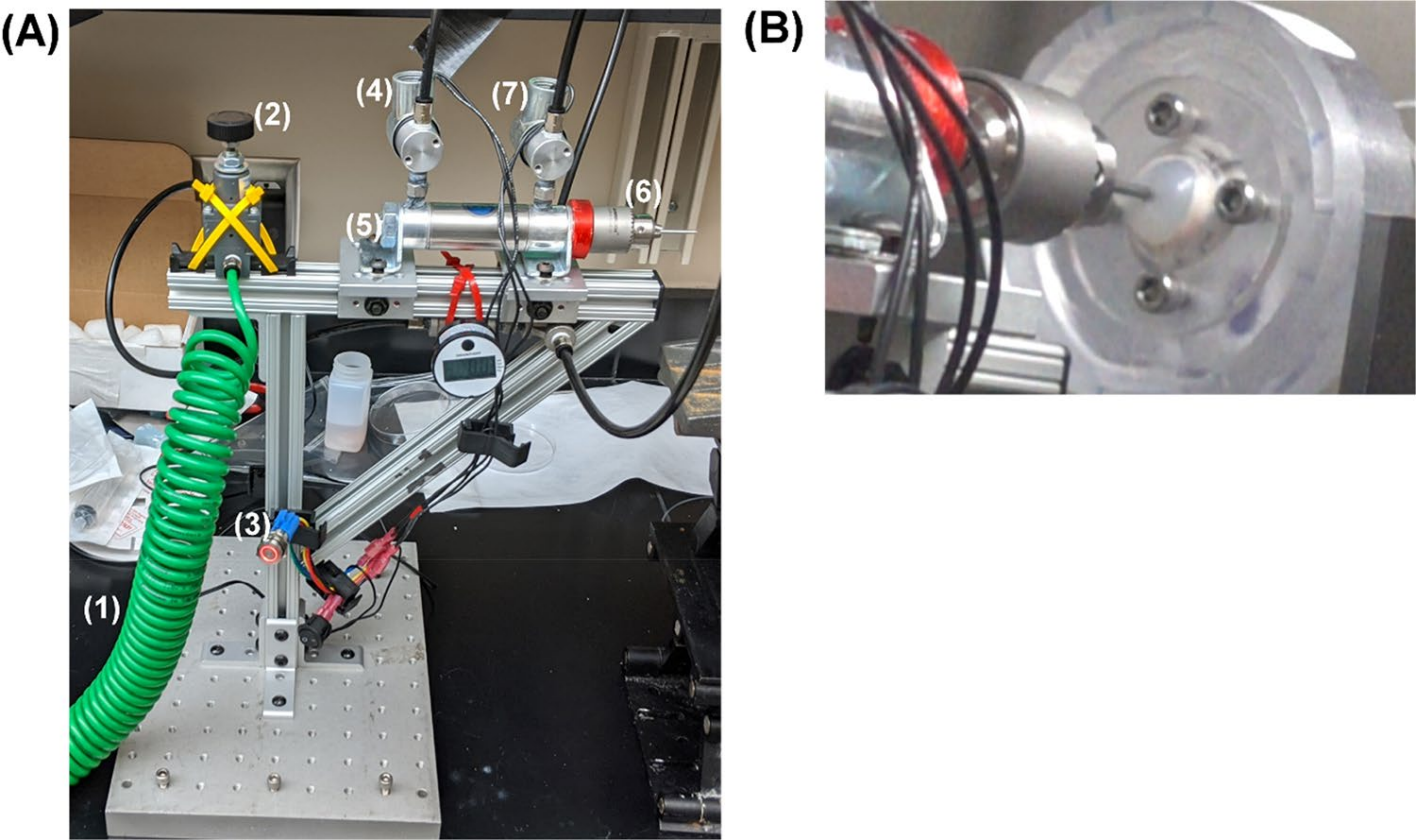
The pneumatic open globe injury device. (**A**) Overview of the device where (1) compressed air is delivered to a (2) pressure regulator set at 50 psi. After the device is (3) fired, the first (4) solenoid valve opens which drives the (5) linear actuator forward causing the (6) drill chuck and puncture object to create an injury in the ASOC setup. Afterwards, the (3) button is released which causes air to be redirected to the second (7) solenoid valve and retract the linear actuator and puncture device from the eye. (**B**) Image of the ASOC setup undergoing OG Injury induction.

We have previously developed a benchtop corneal puncture injury platform that utilizes a solenoid device to create a high-speed injury^11^. To better accommodate work in laminar flow hoods and to make the performance more tunable, we transitioned towards a pneumatic based injury mechanism (Fig. 10A,B). A linear motion piston (Bimba, University Park, IL, USA) is pushed forward with compressed air by opening a solenoid valve (McMaster-Carr, Elmhurst, IL, USA) and is retracted by opening a second solenoid valve that re-directs compressed air to pull the device from the eye. The piston is fitted with a drill-chuck (Llambrich, Barcelona, Spain) to easily accommodate a wide-range of injury sizes. The piston has a travel distance of 3.8 cm when fired. From preliminary testing, it was determined that the corneal apex of the AS needs to be 2.5 cm from the puncture object to create a successful injury. ASOC dish was precisely positioned by holding the dish with a cross-slide vise (McMaster-Carr, Elmhurst, IL, USA). Air pressure was set by pressure regulator (McMaster-Carr, Elmhurst, IL, USA) to 50 psi (~ 2600 mmHg) to ensure successful open OG injury induction. The piston was braced with t-slot framing and mounted to an iron base plate to minimize recoil as the puncture object hits the cornea. Prior to OG injury induction, IOP with in the ASOC dish was set by hydrostatic reservoir at 15 mmHg, and the reservoir was closed prior to firing the puncture device. Throughout, the puncture process was recorded by a high-speed camera (Sony, Tokyo, Japan) to confirm successful OG injury induction.

### Experimental design

**Figure 11:**
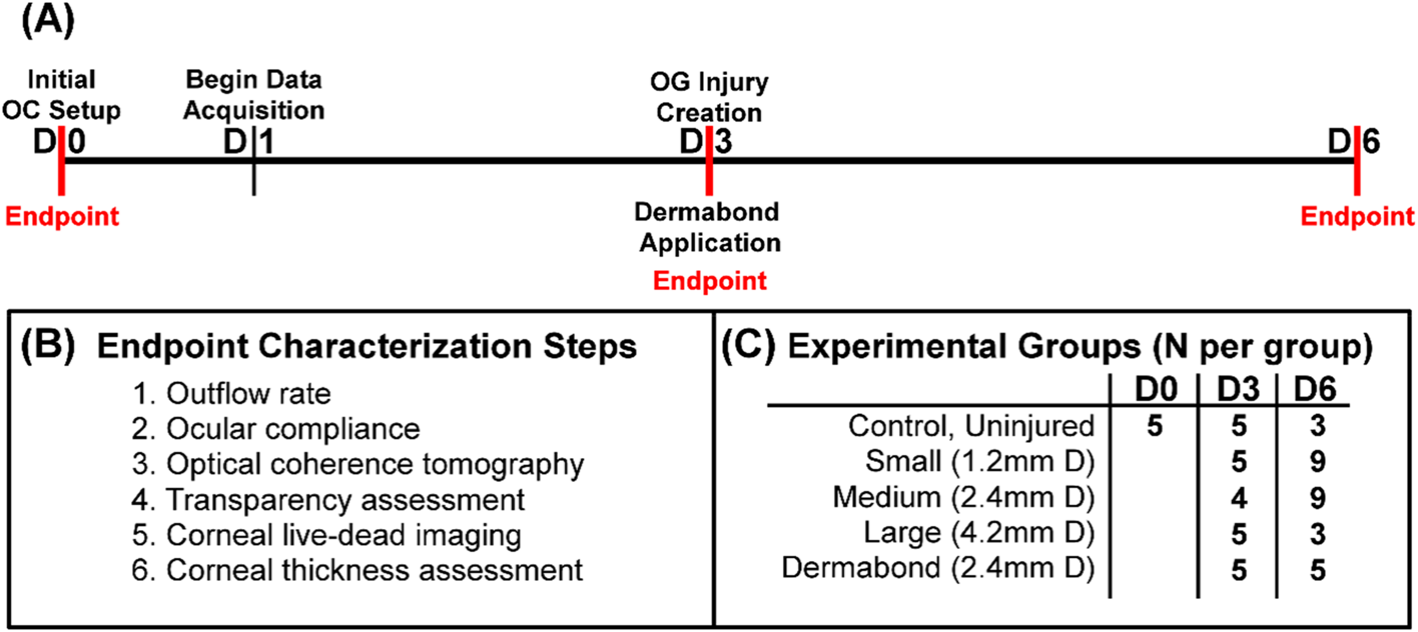
Experiment design for characterizing anterior segment model. (**A**) Timeline for organ culture studies, showing initial setup at D0, initializing data acquisition on D1, creating OG injures on D3, and tracking injury progression out to D6. Endpoint times are indicated in red. (**B**) Overview of the endpoint characterization steps used in this study. (**C**) 11 total experimental groups evaluated in this study which span three time points and 5 experimental conditions. Sample sizes are indicated for the number of ASOC experiments which had IOP values in the acceptable 5 to 20 mmHg IOP range for each group.

For characterizing the OG injury model, eyes were setup at Day 0 and IOP data acquisition was started the next day (Fig. 11A). By Day 3, IOP had sufficient time to stabilize for each ASOC setup, and injuries were induced using the pneumatic puncture device for all injury groups. We then followed injured eyes 72 h post-injury as this was in line with anticipated injured warfighters evacuation delays from the battlefield, and, thus, the initial window an OG injury must be stabilized with a therapeutic. AS tissue was removed from culture and assessed at 3 different timepoints: D0, D3 (post-OG injury), and D6. For endpoint analysis, we measured outflow rate and ocular compliance, imaged the cornea by optical coherence tomography, assessed corneal transparency, evaluated tissue viability by fluorescent imaging, and processed each tissue for histological assessment. More details on each of these methods are described in the following sections (Fig. 11B).

Five experimental conditions were assessed at multiple timepoints for a total of 11 groups (Fig. 11C). Control, uninjured tissue was assessed at Day 0, 3, and 6. Three different injury sizes were evaluated immediately after injury (D3) and 72 h post injury (D6). These injury sizes, 1.2 mm (Small), 2.4 mm (Medium), and 4.2 mm (Large) diameter, were previously evaluated using in vitro test methods and are consistent with military-relevant injury sizes^8,11,32,33^. Lastly, a candidate off-the-shelf tissue adhesive, Dermabond topical skin adhesive (Ethicon, Johnson & Johnson, Somerville, NJ, USA), was applied to medium (2.4 mm diameter) injury sizes. We have previously shown that this therapeutic can create a water-tight seal using the benchtop corneal puncture model^13^. In this study, we assessed wound sealing immediately post-injury (D3) and 72 h post-injury (D6). All studies were performed unmasked.

### Intraocular pressure assessment

Using Arduino-based data acquisition, pressure data were recorded every 10 s for each of up to 10 simultaneous ASOC experiments (1 recording per ASOC dish per second per 10 ASOC dishes). Data acquisition was started 24 h post-initial setup. After removal of eyes from culture on D6, raw readings from pressure data were converted into units of mmHg by calibrating the ten transducers in series using a hydrostatic reservoir to set pressure. Hydrostatic pressure readings were zeroed at the height of the limbus region, approximate location of TM, and increased in increments of 20 cm-H_2_O up to 100 cm-H_2_O for calibration. Pressure vs. time plots were generated for all experiments and stabilized IOP was determined by calculating an average IOP across the 2 to 3 h prior to inducing OG injury. Any eye which did not demonstrate a stable outflow facility (the ratio of perfusion flow rate over IOP, and a measure of TM function) between ~ 0.45 and 0.125 µL/min/mmHg (pressures of ~ 5 mmHg to ~ 20 mmHg IOP) were considered as outliers^17,18^. IOPs for all D6 experiments were averaged 0, 24, 48, and 72 h post-OG injury, by averaging 2 to 3 h of data for each, to determine the effect of injury or adhesive application on stabilizing IOP.

### Ocular mechanics characterization

Ocular compliance was measured by connecting each ASOC dish to an external pressure transducer, syringe pump, and hydrostatic reservoir to record pressure changes, inject volume, and set starting IOP, respectively^11,12^. IOP was monitored by computer data acquisition (LabChart, AD Instruments, Sydney, Australia). Prior to testing each day, system compliance was measured by closing the system from the ASOC dish and isolating the pressure transducer, fluidic connectors, and syringe pump. Volume injections of 20 µL at 20 µL/s were introduced and the increase in pressure was captured. Compliance is a mechanical property defined as the change in volume for a given change in pressure ($\frac{\Delta V}{\Delta P} \left[=\right]\frac{\mu L}{mmHg}$)^34^. We have previously established system compliance standards for this system and any deviation most likely indicates air bubbles trapped in fluidic lines^11^. System compliance measurements were repeated each testing day until values were comparable to system baseline metrics from triplicate measurements.

For each ASOC setup, outflow rate was measured by setting pressure at 18 mmHg and recording the time until pressure decreased to 15 mmHg. We have previously used mathematical models to calculate outflow facility across these data sets, however the injury induction and near instantaneous drops in pressure from the anterior segment created data sets that were impractical to fit to previous developed relationships for calculating outflow facility^11,34^. IOP was set at 18 mmHg using hydrostatic reservoir, the ASOC dish was isolated from the hydrostatic reservoir, and pressure data as a function of time were collected until IOP reached 15 mmHg. This pressure vs. time relationship was evaluated and the duration for the pressure drop to occur was utilized as an indicator of leak rate from the eye.

### Optical coherence tomography

Next, ASOC eyes were imaged by optical coherence tomography (OCT) to visualize the OG injury. Images were taken using a Cirrus HD-OCT system (Carl Zeiss AG, Oberkochen, Germany). Eyes were held at physiological IOP (15 mmHg) during image acquisition by hydrostatic reservoir. For OG injured eyes, images were captured quickly as physiological IOP resulted in PBS leaking from the injury site. Eyes were positioned so the central cornea was in view for uninjured eyes or the OG injury site was central in the image. At least two 3 × 3 mm scans were captured for each eye. Cross sectional video files were generated from each scan, video files were converted to top-down sections, and max intensity projections were created using FIJI^35–37^. Surface area of the injury was quantified using FIJI.

### Corneal transparency assessment

Eyes were removed from ASOC dishes and placed in a custom-printed AS eye holder (Ultimaker 3, Geldermalsen, Netherlands) submerged in saline (anterior cornea side down) on top of 2 × 2 mm grid. Images were captures by digital camera (Sony Corporation, Tokyo, Japan) with calibration ruler in view. Transparency images were semi-quantified using FIJI. First, image files were converted to 8-bit and cropped to center images on the corneal region only. Second, a rolling-ball style background normalization filter was used to minimize local background differences. Third, corneal surface area was measured by selecting the corneal area followed by selecting the area where the underlying grid was visible. A ratio between the visible grid regions and total corneal regions provided a semi-quantitative measurement of corneal transparency.

### Live-dead imaging

Tissue was immersed in Hoechst 33342 (All Nuclei, Thermo Fisher Scientific, Waltham, MA, USA) and ethidium homodimer (Dead nuclei only, Thermo Fisher Scientific, Waltham, MA, USA) following manufacturer’s instructions for 20 min at room temperature. Tissue was left in PBS until imaging by confocal microscopy (LSM 900, Carl Zeiss AG, Oberkochen, Germany). Prior to imaging, scleral tissue was trimmed and the corneal button was place endothelium-side down in a glass bottom petri dish (Electron Microscopy Sciences, Hatfield, PA, USA). PBS was added to submerge the endothelium during the imaging process. 7 × 7 tile scans (10 × objective) were taken centered at the site of injury or central cornea for control tissue. To account for the three-dimensional structure of the eye, a z-stack was taken for all images to capture the endothelium surface. Tiles were stitched and processed as maximum intensity projections across each tiles’ corresponding z-stack of images (Supplementary Fig. 1 for representative images). Images were background normalized, binarized, and signal thresholded followed by quantitating the sum of signal for the All nuclei and Dead nuclei only cell stains, with the difference between the signals corresponding to an estimate of tissue viability.

### Statistical analysis

Five biological replicates were used throughout the experimental groups unless otherwise noted. Replicates were removed from analysis when stabilized IOP fell out of an acceptable range of pressure (5 to 20 mmHg) or if video analysis revealed no OG injury was created for injured groups. All statistical analyses were performed using GraphPad Prism 8.1.2 (La Jolla, CA, USA). Analyses were performed three ways for three sets of data. First, we looked at the effect of ASOC on control tissue which was analyzed for all data sets as a one-way Analysis of Variance (ANOVA) post-hoc Tukey’s test comparing three times points (D0, D3, D6) for uninjured eyes. Second, we looked at the impact of OG injury compared to time matched non-punctured controls at D3 and D6 by two-way ANOVA, post-hoc Dunnett’s test, comparing two time points (D3, D6) for 4 injury states (uninjured, small, medium, large). Third, we looked at the effect of adhesive on an OG injury by comparing time-matched medium OG injury results with and without Dermabond applied at two time points (D3, D6) using a 2-way ANOVA, post-hoc Šídák method. Throughout, asterisks denote (*) *p* < 0.05, (**) *p* < 0.01, (***) *p* < 0.001, and (****) *p* < 0.0001.

## Supplementary Information

Supplementary Information

## Data Availability

The datasets generated during and/or analyzed during the current study are available from the corresponding author upon reasonable request.
