# Supplementary material for: Characterization of an anterior segment organ culture model for open globe injuries
Source: Sci Rep. 2021 Apr 20;11:8546. doi: 10.1038/s41598-021-87910-8 (PMC8058041; doi:10.1038/s41598-021-87910-8)
Supplement: Supplementary file 1 — Supplementary Information [file 41598_2021_87910_MOESM1_ESM.docx]

**Supplementary Information**


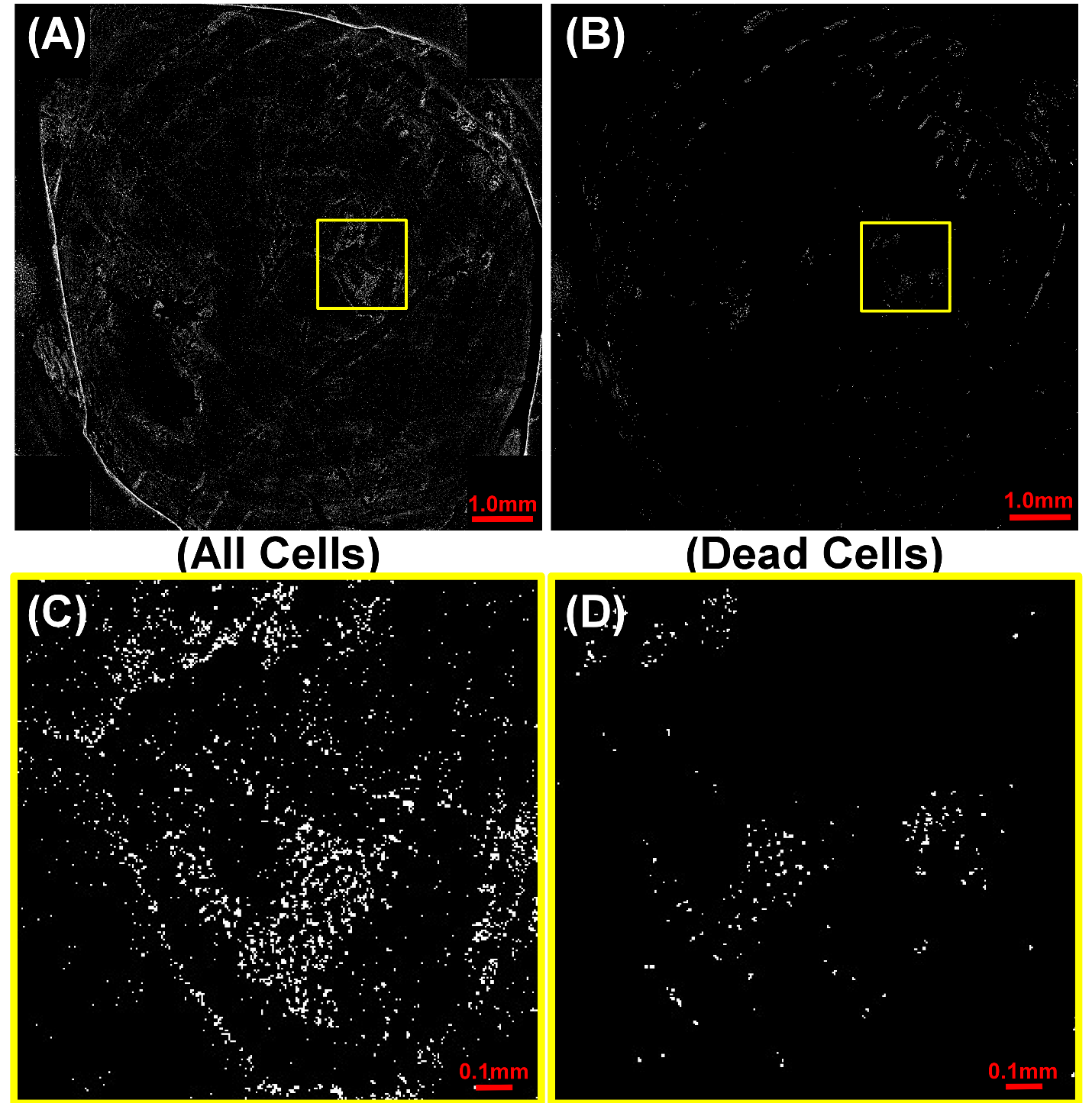


**Supplementary Figure 1: Representative Corneal Viability Micrographs.** Images for a representative Control Day 0 experiment are shown. Micrographs (**A,C**) show the Hoechst 33342 stain for both live and dead stains in the cornea while (**B,D**) Ethidium Homodimer stains only dead cells. Highlighted regions in A and B indicate the zoomed in region shown in C, D. Scale bars are shown for each image.
